# Abundance, Distribution, and Diversity of Freshwater Snail and Prevalences of Their Infection by Cercaria of *Fasciola gigantica* and *Schistosoma* spp at Mayo-Vreck River, Far North Region of Cameroon

**DOI:** 10.1155/2023/9527349

**Published:** 2023-10-20

**Authors:** Augustin Siama, Serges Eteme Enama, Justin Kalmobe, Samuel Abah, Angele Foutchou, Alexandre Michel Njan Nloga

**Affiliations:** ^1^Department of Parasitology and Parasitic Pathology, School of Sciences and Veterinary Medicine, University of Ngaoundere, Ngaoundere, Cameroon; ^2^Department of Animal Biology and Physiology, Faculty of Science, University of Yaounde I, Yaounde, Cameroon; ^3^Special Mission of Tse-Tse Flies Eradication, Ngaoundere, Cameroon; ^4^Department of Biological Sciences, Faculty of Science, University of Ngaoundere, Ngaoundere, Cameroon

## Abstract

Malacological and parasitological studies were conducted from April 2020 to March 2021 to determine the abundance and distribution of molluscs and cercariae of *Schistosoma* spp and *Fasciola gigantica*. Collected molluscs are exposed to strong light to induce cercarial release. Mollusc densities were higher at station 1 (Gamak) than in station 8 (Patakai), with *Bellamya unicolor* and *Biomphalaria pfeifferi* more abundant and *Bulinus truncatus*, *B. tropicus*, and *B. globosus* less abundant. The overall prevalence of cercariae (19.87%) is higher in station 3 (Yaye orchard), station 9 (Gougni), station 4 (Madiogo), station 5 (Madiogo pasture), and station 6 (Ziam 3). It varies significantly between 15.76% in station 8 and 25.77% in station 3, between 8.48% in *B. truncatus* and 25.53% in *B. globosus*, and between 19.27% for cercariae of *Schistosoma* spp and 21.60% for those of *F. gigantica*. Cercarial emissions in *L. natalensis* and *B. pfeifferi* were higher in hot and cold dry seasons; on the other hand, cercarial emissions in *B. globosus* were higher in hot dry seasons (31.48%) and rainy seasons (23.38%). Emissions of cercariae from *S. haematobium* are related to areas of human activity and defecation, while those of *F. gigantica* in *L. natalensis*, *Schistosoma haematobium* in *B. tropicus*, and *S. mansoni* in *B. pfeifferi* are related to grazing areas. Mayo-Vreck is a site that favors the endemicity of fascioliasis and human schistosomiasis.

## 1. Introduction

Freshwater molluscs play a vital role in feeding, recycling nutrients, bioindication of metals, and maintaining water quality [1]. However, some are a real public and veterinary health problem [2, 3] and act as intermediate hosts for parasites [1, 4]. They promote the formation and development of larval stages and the infection of secondary intermediate or definitive hosts [5]. Their impact on the proliferation of waterborne diseases such as hepatic fascioliasis and schistosomiasis is very high [3]. Human schistosomiasis is a neglected tropical disease (NTD) caused by trematodes of the genus *Schistosoma* [6]. Endemic in 78 countries worldwide, it affects more than 229 million people in tropical and subtropical regions, with more than 90% of cases concentrated in sub-Saharan Africa [6, 7]. In the tropics, it is the second most important parasitic disease after malaria in terms of socioeconomic and health impacts [6]. In Africa, *S. mansoni* and *S. haematobium* are widely distributed and are the main cause of human schistosomiasis [7]. Fascioliasis is a parasitic liver disease caused by trematodes of the genus *Fasciola* in herbivorous mammals and humans [8]. It is of great concern due to its high prevalence, its economic importance for livestock in all continents, and its zoonotic aspect [9, 10].

In recent years, cases of fascioliasis and schistosomiasis have been increasing due to climate change, intensification of livestock production and movement, poor environmental sanitation, and the suitability of intermediate hosts to climatic conditions [11, 12]. In the Far North region of Cameroon, the health of human and animal populations is very much affected, especially in Lake Maga, Mayo-Vreck, “yayré” area, and rice fields [13, 14]. Few studies focus on the simultaneous assessment of these diseases at the human/animal-environment-shellfish interface. The present study aims to determine the abundance, distribution, and prevalence of infestation of molluscs by *Fasciola* and *Schistosoma* cercariae in relation to biotic and abiotic factors in Mayo-Vreck. We will more specifically

- determine the abundance and distribution of molluscs
- determine the prevalence and distribution of cercarial infection in molluscs
- assess the impact of biotic and abiotic factors on the distribution of molluscs and cercariae released

## 2. Materials and Methods

### 2.1. Study Site

**Figure 1:**
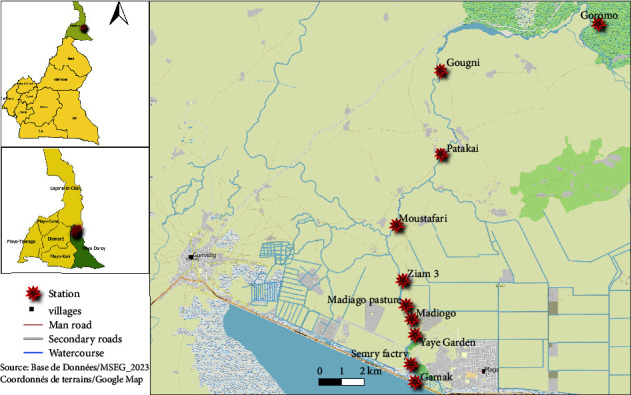
Location of mollusc harvesting sites on the banks of Mayo-Vreck.

Mayo-Vreck (Figure 1) is an old river of Mayo-Tsanaga and Guérléo rivers that supplied “yayrés” and Waza Park with water [15]. Even after the construction of Lake Maga on its bed, improvements were made through the construction of the bridge at Gamak to enable it to play its previous role and serve as an overflow weir for the lake [16]. Populations are settled on both sides of the lake bed, founding neighborhoods and even villages. They practice several activities such as fishing, rice growing, gardening, livestock breeding, and sorghum growing [16]. Mayo-Vreck water is also used to water wild animals in Waza Park, especially in the dry season when most of the ponds are dried up [16]. For the present study, 10 malacological collection stations ranging from Gamak to Goromo localities were selected according to their accessibility and proximity to dwellings, pastures, and areas of human activities. The selected sites were located using GPS to cover the entire river. The distance between two successive sites should be at least 1 km. The 10 malacological collection sites were geolocated in localities such as Gamak (station 1), SEMRY factory (station 2), Yaye orchard (station 3), Madiogo (station 4), Madiogo pasture (station 5), Ziam 3 (station 6), Moustafari (station 7), Patakai (station 8), Gougni (station 9), and Goromo (station 10).

### 2.2. Collection and Identification of Molluscs

Molluscs were collected monthly by two unchanged surveyors during the period from April 2020 to March 2021 using a fishing net or by hand in the waters of the banks or on the plants for 30 minutes following the method of Sarr et al. [17]. These samples, preserved in labelled plastic boxes, were taken to the Maga Fisheries Centre Laboratory for identification. Identification was carried out based on morphological characters such as shell shape and size, shape of the opening, and the number of whorls [18, 19]. After speciation, molluscs are grouped into species and then counted. The monthly count of mollusc species allows us to determine their abundance and the seasonal variation of their populations. Based on the climatic characteristics of the area, the seasons were divided into a rainy season (May to July), a cold dry season (November to January), and a hot dry season (February to April). Harvesting was not possible between August and October due to complete flooding of the sites.

#### 2.2.1. Examination for Trematode Infection

Living molluscs as intermediate hosts of *F. gigantica* and *Schistosoma* spp were washed and placed one at a time in Petri dishes containing distilled water and then exposed to strong photon light for 4–6 hours in order to induce cercarial emission. Each Petri dish is placed under a binocular magnifying glass to observe the cercariae emitted into water. After observation, the cercariae were collected on slides and observed under the microscope for morphological identification according to the identification keys of Frandsen and Christensen [20] and then placed in tubes containing 1.5 ml of 70% ethanol plus 30% distilled water (7 : 3; v/v). The authors only considered the identification of cercariae from *Fasciola* and *Schistosoma* genus. The emission of single cercariae in the mollusc was sufficient to consider it as infected.

#### 2.2.2. Measurements of Physicochemical Parameters and Other Factors

Physicochemical parameters such as pH, temperature of the environment and water, salinity, dissolved solute rate, conductivity, and alkalinity were measured at each visit using a multifunctional pH meter. Rainfall data were taken from the SEMRY II company weather station in Maga. Water depth was determined using a decameter. Plant cover was determined by observing the density of the plant cover. Thus, a score of 0 is given when the site has little or no cover, (1) when the site has little cover, (2) when almost half the site is covered, (3) if more than half the site is covered, and (4) when the site is almost or completely covered by grass. Human activities observed are those that increase the risk of transmission of schistosomiasis and fascioliasis. These include bathing, washing, washing dishes, defecation, watering, breeding, rice-growing, and gardening. To facilitate interpretation, we have grouped all these activities under human activities, with the exception of defecation. Activities were considered at level 0 when no human activity is present, (1) if there is an activity that presents a low risk of contamination (gardening) with a small number of people, (2) in the presence of a high-risk activity carried out by a small number of people (laundry or washing up, bathing, fishing, watering, or rice-growing), (3) if the activity is carried out by several people, and (4) if many of these activities are carried out and concentrated in the same place by several people. Defecation refers to defecation in the open air. The measurements of defecation levels also varied from 0 to 4 according to the intensity of human and animal waste present on the site (0 if there is no feces, 1 if there are less than 5 feces/m^2^, 2 for 5–9 feces/m^2^, 3 for 10–15 feces/m^2^, and 4 for more than 15 feces/m^2^. Proximity to dwellings or distance of dwellings from the watercourse was measured and classified between 0 and 4, with 0 for less than 100 m, 1 for 200–400 m, 2 for 500–700 m, 3 for 800–1000 m, and 4 for more than 1000 m.

#### 2.2.3. Statistical Analysis

The data collected were calculated on the basis of averages and percentages, and their interpretation was carried out using ANOVA, Duncan, chi-square, Schwartz, Simpson, and Shannon tests. ANOVA is used to compare means, the Duncan test is used to compare means, the chi-square test is used to compare percentages, and the Schwartz test is used to rank them. The Simpson and Shannon indices were used to evaluate the specific diversity of the water points of different stations. The abundance of mollusc species (*A* = ni/N) corresponding to the ratio of the total number of individuals of one species (ni) to the total number of individuals of all species (N) [21] and the frequency (Fi) of a mollusc species corresponding to the ratio of the actual presence of a species in all sites (npi) to the number of surveys (Np) times one hundred (*F* = npi/Np *∗* 100) were also calculated.

## 3. Result

### 3.1. Diversity and Abundance of Molluscs

#### 3.1.1. Overall Diversity and Abundance

The fauna of mollusc collected in Mayo-Vreck is composed of 8 species (Table 1) belonging to the class of Pulmonata (*Lymnaea natalensis*, *Bulinus globosus*, *Bulinus tropicus*, *Bulinus truncatus*, *Biomphalaria pfeifferi*, and *Melanoides tuberculata*) and Littorinimorpha (*Bellamya unicolor* and *Bithynia* sp.). The overall count of molluscs during the study period gives 13467 molluscs, with *B. unicolor* (*n* = 3096; *A* = 0.23) and *B. pfeifferi* (*n* = 2987; *A* = 0.22) more abundant, while *B. truncatus* (*n* = 366; *A* = 0.03), *B. tropicus* (*n* = 389; *A* = 0.03), and *B. globosus* (*n* = 560; *A* = 0.04) are less represented. *B. pfeifferi* (*n* = 2987; *A* = 0.22) followed by *L. natalensis* (*n* = 1383; *A* = 0.10) dominates among molluscs of medical and veterinary interest.

#### 3.1.2. Density of Molluscs in the Different Stations

Of the 13,467 molluscs collected (Table 2), the mean densities varied significantly between 59.63 ± 52.62 molluscs in station 8 at Patakai and 468.38 ± 391.7 molluscs in station 1 at Gamak (*F* = 3.12; ndl = 79; *p* < 0.001). Duncan's test shows that the density of molluscs in station 1 is higher than in the other stations. The mollusc species are present in the stations at different frequencies of 100% for *B. pfeifferi*, *M. tuberculata*, and *B. unicolor*, 80%–90% for *Bithynia* sp., *L. natalensis*, and *B. globosus*, and 50% for *B. truncatus* and *B. tropicus*. Simpson's diversity index ranged from 0.689 at station 5 (Madiogo pasture) to 0.814 at station 9 in Gougni. This shows that these stations are very diverse in terms of mollusc species as there is a 68.9%–81.4% chance of collecting mollusc species. Ranking these stations from most to least diverse in mollusc species, we have station 9 at Gougni (*H* = 0.814) > station 4 in Madiogo (*H* = 0.799) > station 1 in Gamak (*H* = 0.798) > station 8 in Patakai (*H* = 0.791) > station 3 in Yaye orchard (*H* = 0.783) > station 7 in Moustafari (*H* = 0, 779) > station 6 at Ziam 3 (*H* = 0.769) > station 10 at Goromo (*H* = 0.740) > station 2 at SEMRY factory (*H* = 0.732) > station 5 at Madiogo pasture (*H* = 0.689).

#### 3.1.3. Spatial Distribution of Stations According to Mollusc Species

**Figure 2:**
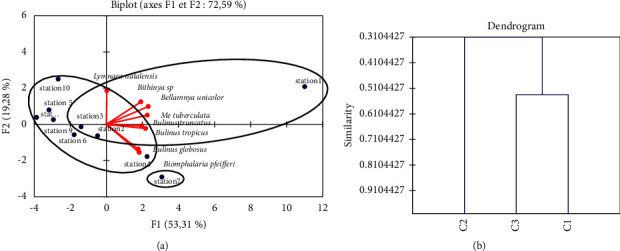
Hierarchical ascending classification (HAC) of stations according to mollusc species. Station 1 = GAMAK; station 2 = SEMRY factory; station 3 = YAYE orchard; station 4 = MADIOGO; station 5 = MADIOGO pasture; station 6 = ZIAM 3; station 7 = MOUSTAFARI; station 8 = PATAKAI; station 9 = GOUGNI; station 10 = GOROMO.

The spatial representation of the harvesting sites according to the abundance and number of species of Mayo-Vreck was analysed using the hierarchical ascending classification (CHA) (Figure 2(a)). The stations analysed in the *F*1 × *F*2 planes are 72.59% visible, while the other axes show only two clouds and one variable. The dendrogram (Figure 2(b)) resulting from the ascending hierarchical classification (CHA) shows that the first cloud is formed by stations *n*° 1–3 (Gamak, SEMRY factory, and Yaye orchard), whereas the second cloud is composed of stations *n*° 4, 5, 6, 8, 9, and 10. Variable III is isolated with only one station (*n*° 7).

#### 3.1.4. Influence of Factors on the Mollusc Population

*(1) Relationship between the Mollusc Population and Seasons*. The results in Table 3 show that the average population densities of *B. truncatus*, *B. unicolor*, and *Bithynia* sp. vary significantly according to seasons ((*F* = 8.49; ndl = 8; *p* < 0.05); (*F* = 7.38; ndl = 8; *p* < 0.05); (*F* = 5.51; ndl = 8; *p* < 0.05)). Duncan's test shows that these species are significantly more abundant during the rainy season and the hot dry season (*p* < 0.05). In contrast, populations of *L. natalensis*, *B. globosus*, *B. tropicus*, *B. pfeifferi*, and *M. tuberculata* did not vary significantly with seasons ((*F* = 4.2; ndl = 8; *p* > 0.05); (*F* = 3.22; ndl = 8; *p* > 0.05); (*F* = 3.55; ndl = 8; *p* > 0.05); (*F* = 3.55; ndl = 8; *p* > 0.05); (*F* = 4.27; ndl = 8; *p* > 0.05)).

*(2) Relationships between Molluscs and Physicochemical Parameters of Water*. The data in Table 4 represent the average values of the physicochemical parameters of the water at the different stations studied. The average alkalinity evaluated at 34.93 ± 3.02 varies between 29.94 ± 1.25 in station 8 and 38.88 ± 3.70 in station 1. The pH evaluated at 7.16 ± 0.53 varies from 6.79 ± 0.31 in station 4 to 7.95 ± 0.59 in station 10. The mean water temperature (T) of the site (26.69 ± 2.04°C) varies between 26.18 ± 1.84°C in station 9 and 28.14 ± 2.98°C in station 10. Conductivity (EC) averaged 206.42 ± 22.8 *µ*S/cm and ranged from 199.99 ± 26.29 *µ*S/cm at station 2 to 211.11 ± 19.09 *µ*S/cm at station 9. The dissolved oxygen rate (DO) evaluated at 6.51 ± 0.46 mg/l is lower in station 9 (6.05 ± 0.30 gm/l) and higher in station 7 (7.14 ± 0.38 mg/l); the water depth (P) evaluated on average at 43.66 ± 28.32 cm oscillates between 27.78 ± 11.99 cm in station 6 and 71.22 ± 41.42 cm in station 10 The dissolved solute content (DSC) averaged 156.98 ± 33.03 ppm throughout the site and is lower in station 2 (140.23 ± 21.37 ppm) and higher in station 10 (185.20 ± 25.48 ppm).

The data in Table 5 represent the correlations between mollusc populations and physicochemical parameters. A positive and highly significant correlation was observed between the *L. natalensis* population and water temperature, while with *B. truncatus*, this correlation was positive and significant ((*r* = 0.439; ndl = 89; *p* < 0.001); (*r* = 0.259; ndl = 89; *p* < 0.05)). The population of *B. pfeifferi* population showed positive and highly significant correlations with temperature (*r* = 0.388; ndl = 89; *p* < 001), negative and significant correlations with water pH (*r* = −0.208; ndl = 89; *p* < 0.05), water depth (*r* = −0.231; ndl = 89; *p* < 0.05), and dissolved solute content (*r* = −0.248; ndl = 89; *p* < 0.05), and a negative and highly significant correlation with conductivity (*r* = −0.258; ndl = 89; *p* < 0.001).

*(3) Relationships between Molluscs and Vegetation and Anthropogenic Activities*. The data in Table 6 representing the correlations between the snail population show the existence of a negative and highly significant correlation between the vegetation cover and the populations of *L. natalensis* (*r* = −0.536; ndl = 89; *p* < 0.001) and *B. truncatus* (*r* = −0.487; ndl = 89; *p* < 0.001). There are negative and highly significant correlations between *B. globosus* (*r* = −0.516; ndl = 89; *p* < 0.001) and *B. truncatus* populations (*r* = −0.524; ndl = 89; *p* < 0.001) with proximity to human habitations, positive and highly significant correlations between *B. globosus* (*r* = 0.40; ndl = 89; *p* < 0.001) and *B. truncatus* (*r* = −0.524; ndl = 89; *p* < 0.001) with proximity to human habitations, positive and highly significant correlations between *B. truncatus* (*r* = 0.40; ndl = 89; *p* < 0.001) and *B. globosus* (*r* = 0.408; ndl = 89; *p* < 0.001) and *B. truncatus* (*r* = 0.590; ndl = 89; *p* < 0.001) populations with proximity to human habitation, and positive and significant correlations between *B. tropicus* (*r* = 0.536; ndl = 89; *p* < 0.05) and *M. tuberculata* (*r* = 0.536; ndl = 89; *p* < 0.05) populations with proximity to pasture. Populations of *B. globosus* (*r* = 0.408; ndl = 89; *p* < 0.01) and *B. truncatus* (*r* = 0.408; ndl = 89; *p* < 0.001) are positively and significantly related to human activities zone such as laundry, washing up, bathing, and watering.

### 3.2. Prevalences of Trematode Cercarial Emissions between Mollusc Species

**Figure 3:**
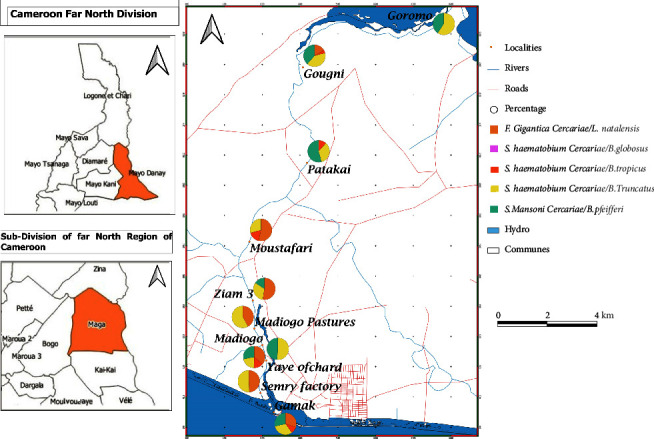
Spatial distribution of cercarial emissions in molluscs.

The information in Figure 3 shows the distribution and the prevalence of cercarial emissions of *F. gigantica* and *Schistosoma spp* in snails from all stations. The cercariae frequently emitted are those of *F. gigantica* in *L. natalensis* and furcocercariae of *Schistosoma* spp including *S. haematobium* emitted by *B. globosus* and *S. mansoni* excreted in *B. pfeifferi*. The prevalences of *S. haematobium* cercariae shed by *B. truncatus* and *B. tropicus* are very low. Overall, cercariae are emitted at the sites in very variable proportions depending on the species and the sampling sites. In total, 2164 out of 5521 molluscs examined emitted cercariae, corresponding to an overall parasitological prevalence of 19.87% (Table 7). This prevalence varied significantly between 15.76% in station 8 and 25.77% in station 3 (*χ*^2^ = 39.95; ndl = 4; *p* < 0.001). The *Z*-test shows that these prevalences are higher in stations 3, 9, 4, 5, and 6. The prevalence of cercariae of *Schistosoma* spp (19.27%) was significantly lower than that of *F. gigantica* (21.60%) (*χ*^2^ = 6.15; ndl = 8; *p* < 0.05). The prevalences of cercariae by mollusc species varied significantly between 8.48% in *B. truncatus* and 25.53% in *B. globosus* (*χ*^2^ = 39.95; ndl = 8; *p* < 0.001). The *Z*-test shows that the prevalences of *S. haematobium* in *B. globosus*, followed by *F. gigantica* in *L. natalensis* and *S. mansoni* from *B. pfeifferi*, are higher.

Cercarial emission rates were globally evaluated at 21.93% and 20.18% in station 1 (Gamak) and 8 (Patakai) and varied significantly between mollusc species ((*χ*^2^_1_ = 17.71; ndl = 8; *p* < 0.05) and (*χ*^2^_8_ = 9.81; ndl = 2; *p* < 0.05)). The *Z*-test shows that cercarial emissions are higher in *B. pfeifferi*, *B. globosus*, and *L. natalensis* in station 1 and in *L. natalensis* in station 8 (Figure 3). In station 7, cercarial emission rates were globally evaluated at 18.47% and vary significantly between mollusc species (*χ*^2^_7_ = 14.26; ndl = 4; *p* < 0.01). The *Z*-test shows that cercarial emission is higher in *B. globosus*. But in stations 2–6 and 9-10, cercarial emission rates did not vary significantly between mollusc species ((*χ*^2^_2_ = 1.86; ndl = 2; *p* < 0.005), (*χ*^2^_3_ = 0.029; ndl = 1; *p* < 0.05), (*χ*^2^_4_ = 9.31; ndl = 3; *p* < 0.05), (*χ*^2^_5_ = 2.41; ndl = 3; *p* < 0.05), (*χ*^2^_6_ = 6.65; ndl = 3; *p* < 0.05), (*χ*^2^_9_ = 7.80; ndl = 4; *p* < 0.05), and (*χ*^2^_10_ = 3.47; ndl = 2; *p* < 0.05)).

The prevalences of *F. gigantica* (21.60%) and *S. haematobium* cercariae in *B. globosus* (25.53%), *B. tropicus* (13.66%), and *B. truncatus* (8.48%) did not vary significantly between stations ((*χ*^2^ = 12.49; ndl = 8; *p* < 0.05); (*χ*^2^ = 8.21; ndl = 7; *p* < 0.05); (*χ*^2^ = 0.52; ndl = 4; *p* < 0.05); (*χ*^2^ = 2.94; ndl = 4; *p* < 0.05), respectively). On the other hand, those of *S. mansoni* in *B. pfeifferi* (20.38%) varied significantly between stations (*χ*^2^ = 25.68; ndl = 9; *p* < 0.001).

### 3.3. Influence of Factors on the Mollusc Population

*(1) Relationship between Cercarial Emissions and Seasons*. The results in Table 8 show overall that the prevalences of cercariae were significantly higher in the cold (20.20%) and hot (24.16%) dry seasons than in the rainy season (14.38%) (*χ*^2^ = 42.74; ndl = 2; *p* < 0.001). The prevalences of *F. gigantica* cercariae in *L. natalensis* and *S. mansoni* in *B. pfeifferi* were significantly higher during the hot (31.05%; 23.03%) and cold (27.38%; 20.49%) dry seasons than during the rainy season (8.71%; 16.72%) ((*χ*^2^ = 55.73; ndl = 3; *p* < 0.001); (*χ*^2^ = 64.08; ndl = 3; *p* < 0.001)), while those of *S. haematobium* in *B. globosus* were higher in the hot dry season (31.48%) and rainy season (23.38%) (*χ*^2^ = 55.73; ndl = 3; *p* < 0.001). The *Z*-test shows that cercarial emissions are significantly higher in the hot dry season in *F. gigantica* and *S. mansoni* and in the hot and then rainy dry season in *S. haematobium*.

*(2) Relationship between Cercarial Emissions and Physicochemical Parameters of Water*. The study of the relationship between cercarial emission and abiotic parameters shows the existence of six significant correlations (Table 9). For water temperature, positive and significant correlations with cercariae of *S. haematobium* in *B. globosus* (*r* = 0.055; ndl = 89; *p* < 0.05) and positive and highly significant correlations with cercariae of *F. gigantica* in *L. natalensis* (*r* = 0.228; ndl = 89; *p* < 0.001) and *S. mansoni* in *B. pfeifferi* (*r* = 0.228; ndl = 89; *p* < 0.001) were observed. Highly significant and positive correlations were observed between the emission of cercariae of *F. gigantica* in *L. natalensis* (*r* = 0.240; ndl = 89; *p* < 0.0001) and of *S. mansoni* in *B. pfeifferi* (*r* = 0.237; ndl = 89; *p* < 0.001) with conductivity. Only one positive and highly significant correlation was observed between the emission of *S. mansoni* cercariae in *B. pfeifferi* and alkalinity (*r* = 0.069; ndl = 89; *p* < 0.012).

*(3) Relationships between Cercarial Emissions and Anthropogenic and Environmental Factors*. The results in Table 10 show the existence of 14 significant correlations between cercarial emission rates and anthropogenic and environmental factors. The emission of *F. gigantica* cercariae in *L. natalensis* and *S. haematobium* cercariae in *B. truncatus* is negatively and highly significantly correlated with plant cover ((*r* = −0.573; ndl = 89; *p* < 0.001); (*r* = −0.304; ndl = 89; *p* < 0.01)). *S. haematobium* cercarial emissions in *B. globosus* and *B. truncatus* were negatively and highly significantly correlated to human habitation areas ((*r* = −0.391; ndl = 89; *p* < 0.001); (*r* = −0.300; ndl = 89; *p* < 0.01)) but are positively and highly significantly correlated to areas of human activity ((*r* = 0.212; ndl = 89; *p* < 0.05); (*r* = 0.298; ndl = 89; *p* < 0.01)). Cercarial emissions rates of *F. gigantica* in *L. natalensis*, *S. haematobium* in *B. tropicus*, and *S. mansoni* in *B. pfeifferi* were significantly related to grazing areas ((*r* = 0.226; ndl = 89; *p* < 0.05); (*r* = 0.270; ndl = 89; *p* < 0.01); (*r* = 0.257; ndl = 89; *p* < 0.01)), whereas emissions of *S. haematobium* in *B. globosus* and *B. truncatus* are significantly related to it ((*r* = 0.281; ndl = 89; *p* < 0.007); (*r* = 0.401; ndl = 89; *p* < 0.001)). *S. haematobium* cercarial emissions in *B. globosus*, *B. tropicus*, and *B. truncatus* are positively and highly significantly correlated to defecation areas ((*r* = 0.404; ndl = 89; *p* < 0.001); (*r* = 0.297; ndl = 89; *p* < 0.004); (*r* = 0.337; ndl = 89; *p* < 0.001)).

## 4. Discussion

Of the molluscs collected, six of eight species are of medical and veterinary importance from their role as host intermediates for human and animal parasites. These results are similar to those of Igbinosa et al. [21], Oloyede et al. [22], Oladejo et al. [23], and Siama et al. [24] in the Ova ecosystem, Eleyele Dam, and Ogunpa River in Nigeria and the Douvar Reservoir in Cameroon. In contrast, El-Zeiny et al. [25] reported a predominance of species of no medical or veterinary importance in Damietta, Egypt. These results reflect the importance of watering holes in the epidemiology of molluscan vector-borne diseases in the Sahelian zone [3, 26].

The overall count of molluscs from all stations estimated at 13467 individuals' shows that *B. unicolor* and *B. pfeifferi* are more abundant, while *B. truncatus*, *B. tropicus*, and *B. globosus* are less so. Of the species of medical and veterinary interest, *B. pfeifferi* followed by *L. natalensis* is the most abundant. Amawulu and Assumpta [27] and Dida et al. [8], on the other hand, reported an abundant population of *L. natalensis* compared to *B. globosus*, *Pila* sp., *Melanoides* spp, and *B. forskalii* in the Niger Delta of Nigeria and the Mara River Basin of Kenya and Tanzania. Dogara et al. [28] observed high abundance of *B. globosus* in the Warwade Dam in Nigeria. According to Pedersen et al. [29], climatic conditions and characteristics of aquatic environments play an important role in the distribution of molluscs. The abundance of *Biomphalaria* spp could be justified by their preference for permanent water bodies [1, 30], while *Bulinus* spp are less abundant due to their strong preference for seasonal water bodies [1, 31].

The density of molluscs at station 1 in Gamak is greater than at the other stations because it opens directly to Maga Lake via a bridge and is less overgrown. Their abundance at this station is also thought to be related to the important human activities of defecation, micturition, and washing, which favors their development by increasing the growth and abundance of algae recognized as their best food [32, 33]. *B. pfeifferi*, *M. tuberculata*, and *B. unicolor* are very frequent in all stations (100%), *Bithynia* sp., *L. natalensis*, and *B. globosus* are frequent in most stations (80%–90%), and *B. truncatus* and *B. tropicus* are found in half of the stations (50%). These observations show that these sites are favorable to the survival of most mollusc species due to the permanence of water, which according to Bakhoum et al. [34] is an important factor in the distribution of molluscs. Simpson's diversity index ranging from 0.689 in station 5 (Madiogo pasture) to 0.814 in station 9 (Gougni) shows that these stations are very diverse in species as there is a 68.9–81.4% chance of encountering several species of snails. These results are similar to those of El-Zeiny et al. [25] in Damietta, Egypt, but for Salawu and Odaibo [35] in Yewa, Nigeria, it would be an indication of the stable coexistence of molluscs in the same ecosystem According to El Deeb et al. [36], several factors such as the use of agricultural chemicals, the degree of pollutants, and water velocity can affect the density and distribution of molluscs in the beds. The dendrogram from the bottom-up hierarchical classification (BHC) shows a distribution of stations according to mollusc species in two clouds and one variable. In cloud 1, stations share 5 species (*L. natalensis*, *B. pfeifferi*, *M. tuberculata*, *B. unicolor*, and *Bithynia* sp.), the average population densities of *B. pfeifferi*, *Bithynia* sp., and *B. unicolor* are higher, and their species richness is high (5–8 species). The stations in cloud 2 share four species (*L. natalensis*, *M. tuberculata*, *B. unicolor*, and *Bithynia* sp.) with very high population densities. Their species richness is also high (6–8 species). In variable III, station 7 is distinguished by the total absence of *L. natalensis* and the dominance of *B. pfeifferi* and *Bithynia* sp. In contrast to the other clouds, *B. globosus*, *B. tropicus*, and *B. truncatus*, less abundant in the other stations, are more abundant in this station.

Populations of *B. truncatus*, *B. unicolor*, and *B. unicolor* are more abundant during the rainy and warm dry seasons, while those of *L. natalensis*, *B. globosus*, *B. tropicus*, *B. pfeifferi*, and *M. tuberculata* are important without varying significantly between seasons. These results are similar to those of El-Kady et al. [37] who reported high densities of molluscs in April in the Sinai Peninsula, Egypt. But significant variations in densities were reported in May for *P. marmorata*, September/October for *L. natalensis*, June for *B. pfeifferi* and *B. globosus*, June/July for *M. tuberculata*, and August/October for *I. exutus* in Igwun waters of southwestern Nigeria by Owojori et al. [38]. In addition, Ofoezie [39] and Owojori et al. [38] in the Eleyele Dam and in rivers and ponds in Ile-Ife and Oyan in Nigeria and Manyangadze et al. [40] in Ingwavuma in South Africa reported higher numbers of gastropods in the rainy season. These results show that climate exerts a major influence on the geographical distribution of snails through the characteristics of water bodies [41]. Precipitation is also a limiting factor in mollusc abundance as the habitat of molluscs can be affected by precipitation, which cannot survive without water, but too much water at velocities above 0.3 m/s also reduces their populations [40, 42, 43].

The observed correlations between water temperature and populations of *B. truncatus*, *Bithynia* sp., *L. natalensis*, *B. pfeifferi*, *B. unicolor*, and *M. tuberculata* are in agreement with those of Kazibwe et al. [44] conducted in Lake Albert, Uganda, and Hussein et al. [45] in Qena Governorate, upper Egypt, who, respectively, reported a positive relationship between the abundance of *Biomphalaria* sp., *B. unicolor*, and *L. carintus* with water temperature. On the other hand, Owojori et al. [38] reported a negative significant relationship between temperature and *B. globosus* densities in the Eleyele Dam in Nigeria. On the other hand, Ofoezie [39] reported no significant relationship between mollusc densities and temperature in Nigeria. Alkalinity influencing the populations of *B. unicolor*, *Bithynia* sp., and *M. tuberculata* could be explained by the organic pollution of the stream. According to Adekiya et al. [33], most snails are abundant in waters polluted by human excreta and sewage from domestic waste where algae abound. Densities of population of *B. pfeifferi* and *B. unicolor* are negatively influenced by water pH, conductivity, water depth, and TDS. Regarding pH, Ofoezie [39] and Cañete et al. [46] found no significant relationship between mollusc abundance and pH. However, Owojori et al. [38] recorded a positive correlation between pH and *Potadoma* sp., *C. bulimoides*, *M. tuberculata*, and *T. niloticus*, while Ernould et al. [47], Hussein et al. [45], and El Deeb et al. [36] reported negative correlations between pH and *Biomphalaria* sp. According to Owojori et al. [38], Monde et al. [48], and Olkeba et al. [49], the pH tolerance limits of most species of snails in natural water bodies are inside the range of 5.0–9.0. However, some mollusc intermediate hosts of *Schistosoma* have been observed at pH values lower than 4.0, although this is theoretically impossible [29]. Regarding the influence of conductivity, Owojori et al. [38] reported a positive relationship between conductivity and *P. marmorata*, *B. globosus*, and *P. liberiensis* in the Eleyele Dam in Nigeria. Regarding the negative influence of depth, Hussein et al. [45] in Egypt also observed a negative relationship between water depth and *G. africana*, *B. pfeifferi*, and *B. globosus* and also a positive relationship with *P. marmorata*. Most studies indicate a negative correlation between river depth, river width, and abundance of mollusc species [31, 45]. The preference of these molluscs for shallow water seems to be correlated with light availability [50]. According to Lydig [51], prolonged artificial shading for 6 weeks influences the *B. pfeifferi* population through its effect on food sources. The association between *B. truncatus* abundance and high algal densities, macrophytes, and substrate parameters support this conclusion [45]. On the other hand, molluscs are less numerous in steep shores than in gentle slopes. However, in large water bodies, waves exceeding 10 cm can significantly influence the *Bulinus* spp population on gentle slopes [52]. *B. pfeifferi* is less resistant to high waves, which explains its preference for small pools and sheltered areas [53]. Sediments may contain small particles and have large food items that favor the survival and extensive multiplication of molluscs [23, 34]. The results on the significant relationship between dissolved oxygen and *B. unicolor* differ from those of Owojori et al. [38] and Hussein et al. [45] who recorded no significant correlation between snail density and dissolved oxygen. However, Idowu et al. [54], Mereta et al. [42], and Ismail et al. [55] observed an increase in mollusc population with that of dissolved oxygen. According to Sunita et al. [56], dissolved oxygen is an important element for the survival of molluscs in the aquatic environment.

Vegetation cover negatively and significantly influences the populations of *L. natalensis* and *B. truncatus*. Oleyede et al. [22] also showed that *L. natalensis* and *Ceratophallus* spp prefer open rock pools and stream margins, while *Biomphalaria* spp and *B. africanus* prefer habitats with more abundant and constant vegetation. However, aquatic and marginal plants provide an oviposition environment and food for freshwater snails and support the growth of populations such as *Biomphalaria* [8]. Human habitations are negative and highly significant close to that of *B. globosus* and *B. truncatus* populations. These results differ from those of Oleyede et al. [22] who observed no significant difference in the Eleyele Dam in Nigeria. In Tanzania, Lydig [51] reported that populations of *Biomphalaria* spp, *Bulinus* spp, and *Lymnaea* spp are significantly abundant in the vicinity of human settlements in Babati district. These results could be explained by seasonal fluctuations in the bed of Mayo-Vreck and changes in activities that cause people to move away from the banks to avoid flooding in the rainy season. *B. globosus*, *B. truncatus*, and *M. tuberculata* are distributed significantly close to areas of human activity. These observations corroborate with those of Dida et al. [8]. In contrast, Oleyede et al. [22] and Amawulu et al. [57] reported that human activity showed no significant effect on snail distribution. Ernould et al. [47] also reported that in the irrigated perimeters of Niger, the distribution of bilharzian risk appears to be closely related to the proximity of the habitat to secondary irrigation canals. Furthermore, according to Marie et al. [58], Calasans et al. [32], and Adekiya et al. [12], human activities such as defecation, washing, micturition, and sewage waste favor the survival and reproduction of molluscs as they increase the growth and abundance of their best food items such as algae. *B. globosus* and *B. truncatus* are more related to grazing areas than *B. tropicus* and *M. tuberculata*. Bakhoum et al. [43] reported that distribution of *B. globosus, B. truncatus*, *B. tropicus*, and *L. natalensis* around human habitats would contribute to increased risks of transmission of fascioliasis and schistosomiasis.

The overall prevalence of trematode cercariae infections in snails at 19.87% is higher than that reported by Baldwin et al. [59] in the Omo Gibe riverbanks in Ethiopia (3.6%), Devkota et al. [60] and Pandey [61] in Chitwan (3.5%) and Kavre (1.7%) districts in Nepal, Ngonseu et al. [62] in intermediate hosts of schistosomes in Cameroon (0.08%), and Steinauer et al. [63] in Lake Victoria (1.04%). However, Olkeba et al. [52] reported a higher prevalence in the Rift Valley lake (30.5%). This difference could be justified by the high contamination of the water by human or animal feces and urine containing the eggs of these parasites. According to Bekana et al. [26], intermediate host molluscs play a crucial role in locating sensitive areas for schistosomiasis transmission. The cercarial emission rates are higher in stations 10 in Dawaya, 4 in Madiogo, 5 in Madiogo pasture, and 6 in Ziam 3. These results can be explained by the proximity of pastures and dwellings to human activities and human defecation areas. According to Tchuem Tchuenté et al. [7] and Adekiya et al. [33], levels of schistosomiasis endemicity vary with the initial endemic level, the distance between the dwelling and potential transmission sites, and the sociological phenomena that link humans with the contamination sites. The overall prevalences of cercariae are highest in *B. globosus* followed by *L. natalensis* and *B. pfeifferi*. These observations are similar to those of Kinanpara [64] in Côte d'Ivoire, who reported higher prevalences in *B. globosus* than in *B. pfeifferi*. In contrast, Moser et al. [65] reported higher prevalences in *B. forskalii* (36.4%) than in *B. globosus* (34.8%) and *B. pfeifferi* (0.9%) in Chad. This result confirms the observations of Saotoing et al. [13] who reported a predominance of *S. haematobium* urinary tract infection among students in the town of Maga. According to Hotez and Kamath [66] and Degarege et al. [67], in sub-Saharan Africa, more than 112 million cases of schistosomiasis are urogenital caused by *S. haematobium*, representing about 50% of the total incidence of *Schistosoma* infection. This may be largely due to the wide geographical distribution of *Bulinus* spp host intermediates [12, 33, 68]. High contamination of molluscs could be explained by the urination of humans and domestic and wild animals into the watercourse, as during contact with water, infected humans or animals promote the release of *S. haematobium* eggs [33, 47]. In stations 1 (Gamak), 8 (Patakai), and 7 (Moustafari), cercarial emissions were significantly higher in *B. pfeifferi*, *B. globosus*, and *L. natalensis* in station 1, in *L. natalensis* in station 8, and in *B. globosus* in station 7 at 31.93%, 40.61%, and 38.02%, respectively. The predominance of *S. mansoni* cercariae in *B. pfeifferi*, *S. haematobium* in *B. globosus*, and *F. gigantica* in *L. natalensis* in station 1 reflects the important role that hosts play in the endemicity of schistosomiasis and fascioliasis. Indeed, Vreck, which is linked to Lake Maga by a bridge, serves as an overflow and irrigation channel for the waterways of Waza Park in the dry season. Human populations are settled on both sides of the riverbed and carry out activities there (fishing, agriculture, livestock breeding, washing, etc.). This proximity to the site accentuates water-human and water-animal contacts and increases the risk of schistosomiasis and fascioliasis. According to Pedersen et al. [29], the density of humans/animals around the sites directly conditions the richness of water in miracidium through their micturition and defecation in the aquatic environment, which increases the likelihood of reinfestation through human/animal-water contact. In station 8, the predominance of *F. gigantica* cercariae is thought to be related to the nature of the environment, which is conducive to animal grazing in the dry season and to the survival of *L. natalensis* populations. According to Saotoing et al. [69] and Hailegebriel et al. [3], the proximity of watercourses to grazing land favors permanent miracidium-mollusc contact through animals and an increase in cercarial emission rates and frequencies. The predominance of *S. haematobium* cercariae in station 7 at Moustafari is thought to be due to the large population of *B. globosus* and intense human activities. Indeed, these populations, lacking means to dig deep boreholes, use the river water for drinking, washing themselves, doing their laundry and dishes, and watering their animals. On the other hand, eggs hatch and release miracidia in water that will penetrate specific snail [11, 70].

The prevalence of *F. gigantica* cercariae estimated at 21.60% in *L. natalensis* is higher than that reported by Iglesias-Piñeiro et al. [10] in Spain (4.4%) and Mekonnen et al. [71]. In contrast, Muñoz-Antoli et al. [72] obtained higher prevalences (44.72%) in *L. natalensis*. These differences could be explained by variation in ecosystems and mollusc-cercaria-animal relationships [73–75]. The high prevalences in stations 1 (Gamak), 3 (Patakai orchard), 4 (Madiogo), and 8 (Patakai) are thought to be related to the proximity to pasture and stream and the presence of low vegetation cover. Infected cattle shed *F. gigantica* eggs in their feces and help maintain the fasciolian risk.

The prevalence of *S. haematobium* cercariae estimated at 25.53% in *B. globosus* is higher than that obtained by Dabo et al. [76] in Mali (7.8%), Ayanda [77], Iboh et al. [78], Aliyu et al. [79], and Afiukwa et al. [4] in Nigeria (18.37%; 10.8%; 24.30%; 19.2%), Opisa et al. [80] in Kenya (2.2%), Kinanpara et al. [64] in Côte d'Ivoire (12.17%), and Allan et al. [81] in Tanzania (14.5%), and Mutsaka-Makuvaza et al. [82] in Zimbabwe (1.9%). However, higher prevalences were obtained by Moser et al. [65] in Chad (34.8%), Abe et al. [83] in *B. truncatus* (44.64%), Akinwale et al. [84] in *B. camerunensis* (57%) in Nigeria, and Moser et al. [65] in *B. forskalii* (36.4%) in Chad. The high prevalences observed in Gamak, Madiogo, and Moustafari stations could be explained by proximity to the population, the intense human activity in the river, and water-human contact as reported by Bakhoum et al. [43]. In *B. pfeifferi*, the prevalence of *S. mansoni* cercariae estimated at 20.38% is higher than that reported by Alebie et al. [85], Alemayehu and Tomass [86], and Amsalu et al. [87] in Ethiopia (10.6%, 3.1%, and 6.3%), and Olkeba et al. [49] in Nigeria (20.31%) and lower than that reported by Ayanda [77] in Nigeria (30.5%), Fuss et al. [30] in Tanzania (35.4%), and Mengistu et al. [88] and Bekana et al. [26] in Ethiopia (58%; 24.4%). The high prevalences of cercariae observed in stations 10 (Goromo), 1 (Gamak), and 3 (Yayé garden) near human settlements and pastures show their importance in maintaining the schistosome cycle. According to Bekana et al. [26], the risk of cercarial infections is conditioned by the contamination of water by human feces containing eggs, the availability of host molluscs, aquatic birds, and the presence of domestic and/or wild animals. Children's behavior in terms of frequency and duration of water contact, environmental exposure, and social and cultural practices are thought to contribute to the maintenance of shellfish infection, as they are more likely to spend time around cercarial-infested water [32, 33]. Other sources of contamination may include washing of fecally contaminated clothes, cleaning of the perianal area after defecation, and excreta from reservoir hosts such as wild animals [89].

The cercarial emissions in overall *Fasciola* and *Schistosoma* species higher in the hot (24.16%) and cold (20.20%) dry seasons than in the rainy season (14.38%) could be explained by epidemiological factors such as climate change characterized by flooding and vegetation development in the rainy season, which contribute to the dispersal and decrease in mollusc quantity and to limiting human-mollusc contact [49, 90, 91]. Climate change thus indirectly affects cercarial emission and successful penetration on definitive hosts. Shellfish predators also affect cercarial emission rates when decreasing shellfish numbers. Competitors limit development in the intermediate host, reducing the number of cercariae [49, 90, 91].

The cercarial prevalence of *F. gigantica* in *L. natalensis* is higher in the hot (31.05%) and cold (27.38%) dry seasons than in the rainy season (8.71%). On the other hand, Islam et al. [9] observed that the prevalences of gymnocephalus cercariae emerging from *Lymnaea* sp. vary significantly with seasonal changes, reaching a peak between April and October, then decreasing from February to March, and disappearing between November and January. According to Qureshi et al. [92], cercariae can be observed in large numbers on vegetation during the rainy season and at the beginning of the dry season along the banks of rivers, lakes, and streams. However, Smith [41] studying predictive models in France under two different greenhouse gas emission scenarios showed that the population of *F. hepatica* will increase dramatically in the future, with more than double the number of cercariae currently observed between August and October and significantly more contamination of pastures between June and December. The difference in ecology and rainfall regime could contribute to the difference observed in our study area. The infection rate of *Biomphalaria* by *S. mansoni* is higher during the hot (23.03%) and cold (20.49%) dry seasons than during the rainy season (16.72%). Similar results have been reported from Tanzania [93], Sudan [55, 94], and Nigeria [95], indicating that schistosomiasis-infected *B. pfeifferi* molluscs were elevated during a dry season. Bekana et al. [26] reported higher *B. pfeifferi* infection rates in Ethiopia (28.9%) after the rainy season (October to December), followed by the dry season between January and March (12.3%), while no infected snails were observed during the rainy season from June to September. Rainfall, open defecation levels, human-water contact activities, and stable water conditions during dry seasons could contribute to the long-term survival of *Biomphalaria* populations, resulting in a high risk of infection by *S. mansoni* cercariae [26, 96]. The higher emission rates of *S. haematobium* cercariae in *B. globosus* in the hot dry season (31.48%) and rainy season (23.38%) differ from the results of Saathof et al. [97] who reported that at uMkhanyakude in South Africa, *B. globosus* excreted cercariae in all seasons with the highest proportion in the rainy season. In Ethiopia, Xue et al. [98] found that rainfall may be responsible for increasing the population dynamics of schistosomes through the accumulation of sufficient surface water in ponds but also causes water turbulence that increases their flow, which in turn disrupts snail habitats and decreases the survival capacity of cercariae. In sub-Saharan Africa, Ernould et al. [70] and Hailegebriel et al. [3] observed an increase in the transmission of *S. mansoni* cercariae by *B. pfeifferi* compared to *S. haematobium* by *B. globosus* during rainy periods on the banks of the Senegal River, suggesting an increase in the transmission of *S. haematobium* during the dry period.

Significant relationships between cercarial emissions of *F. gigantica* in *L. natalensis*, *S. haematobium* in *B. globosus*, and *S. mansoni* in *B. pfeifferi* and water temperatures have also been reported by Yirenya-Tawiah et al. [99], Islam et al. [9], Marie et al. [58], and El Deeb et al. [36] in several African countries. Islam et al. [9] revealed that the water temperature required for the release of gymnocephalus cercariae in *Lymnaea* sp. ranged from 18 to 34°C. According to McCreesh and Booth [100], temperature influences the speed of miracidia as they penetrate snails, as well as the release of cercariae from molluscs and their penetration of the skin of the final host. The release of cercariae from *F. gigantica* in *L. natalensis* and from *S. mansoni* in *B. pfeifferi* was significantly and positively correlated with conductivity. These results do not agree with those of Sunita et al. [56] who reported negative correlations between the prevalence of gymnocephalus and xiphidiocercariae cercariae in *L. natalensis* and *B. truncatus* and conductivity. Mereta et al. [42] found that the prevalence of xiphidiocercariae in *L. natalensis* was negatively correlated with water conductivity. According to Poulin and Mouritsen [101], the effect of conduction on cercarial release is due to ionic changes in water caused by light and temperature that favor cercarial release in host molluscs. However, further studies are needed to support this argument.

The greater the cercarial release from *F. gigantica* in *L. natalensis* and from *S. haematobium* in *B. truncatus*, the lower the vegetation cover. These observations, similar to those of Johnson and Paull [91] and Okelba et al. [49], would be justified by the distribution of intermediate host molluscs, which are also less numerous as vegetation density increases. The levels of *S. haematobium* cercariae in *B. globosus* and *B. truncatus* decrease very significantly the closer they are to human habitations, but they increase when approaching areas of human activity. In areas close to grazing land, emissions of *F. gigantica* cercariae in *L. natalensis*, *S. haematobium* in *B. tropicus*, and *S. mansoni* in *B. pfeifferi* are high, while those of *S. haematobium* in *B. globosus* and *B. truncatus* are very high. According to Hailegebriel et al. [11], human activities such as open defecation, urination, livestock grazing, agriculture, and swimming are strongly correlated with trematode infection. In these areas, aquatic habitats are commonly used for open defecation and urination, washing, bathing, and animal watering. These practices can result in the release of schistosome eggs through urine, which after hatching release miracidia and enter the host snails to produce cercariae [102]. The positive and significant correlations between the release of *S. haematobium* cercariae in *B. globosus*, *B. tropicus*, and *B. truncatus* and defecation are surprising, as one would expect the high impact of *S. mansoni* cercariae released in feces. This difference could be explained by the easy dispersal of *S. haematobium* eggs at the expense of *S. mansoni* [26]. According to Saotoing et al. [69], *S. haematobium* eggs are released directly during urination in urine and discharged directly into water, while *S. mansoni* eggs must first undergo a complete dilution of feces for their full release before being disseminated afterwards. In addition, the human habit of defecating in bushes or at the water's edge does not directly facilitate this dilution [26].

## 5. Conclusion

Mayo-Vreck is a site that is very diverse and rich in mollusc species such as *L. natalensis B. globosus*, and *B. pfeifferi* which play an important role in the transmission of waterborne diseases such as fascioliasis and human schistosomiasis in the locality of Maga and its surroundings. The prevalences of cercariae in these molluscs are high, especially in *L. natalensis, B. globosus*, and *B. pfeifferi.* The distribution of molluscs and the prevalence of cercariae around habitats and areas of human activity provide sufficient information on the epidemiological character and the high risk of transmission of these diseases among the local population. They also show that prevention campaigns using praziquantel among schoolchildren and antihelmintic drugs among domestic animals are not enough to eradicate the diseases they cause. A synergy of action through the fight against intermediate host molluscs and education and awareness campaigns for the population on the modes of transmission of these diseases and the means of their prevention, such as limiting bathing, defecation, and micturition in contaminated water, could help solve the problem. In the future, it would be wise to determine the incidence of the disease on the local populations and to evaluate different strategies they adopt to fight against these waterborne diseases.

## Figures and Tables

**Table 1:** Diversity and abundance of molluscs in Mayo-Vreck.

Class	Pulmonata						Littorinimorpha		*Total*
Family	Lymnaeidae	Planorbidae				Thiaridae	Viviparidae	Bithyniidae	
Subfamily		Bulininae			Planorbinae				
Species	*L. natalensis*	*B. globosus*	*B. truncatus*	*B. tropicus*	*B. pfeifferi*	*M. tuberculata*	*B. unicolor*	*Bithynia* sp.	
May	272	61	39	46	295	299	409	353	1774
June	192	61	37	41	293	217	312	227	1380
July	121	79	52	51	345	223	353	282	1506
November	24	46	18	26	177	155	163	106	715
December	96	40	28	29	260	236	231	145	1065
January	121	57	33	37	290	231	283	170	1222
February	167	57	44	36	368	262	319	239	1492
March	214	90	55	54	415	366	469	333	1996
April	176	69	60	69	544	454	557	388	2317

Total (*n*)	1383	560	366	389	2987	2443	3096	2243	13467
Means	153.67	62.22	40.67	43.22	331.89	271.44	344	249.22	
Standard deviation	72.51	15.47	13.55	13.43	104.38	90.05	120.52	97.71	
Abundance (*A*)	0.10	0.04	0.03	0.03	0.22	0.18	0.23	0.17	

**Table 2:** Diversity and density of malacological fauna in different stations.

Stations	Species locality	*L. natalensis*	*B. globosus*	*B. truncatus*	*B. tropicus*	*B. pfeifferi*	*M. tuberculata*	*B. unicolor*	*Bithynia* sp.	Total	Mean ± standard deviation	*H*′	*D*′
1	Gamak	218	101	136	136	390	900	808	1058	3747	468.38 ± 391.7a	1.769	0.798
2	SEMRY factory	11	42	00	00	293	68	324	334	1072	134.0 ± 153.64b	1.428	0.732
3	Yaye orchard	151	00	00	00	206	102	251	242	952	119.0 ± 109.49b	1.562	0.783
4	Madiogo	184	154	00	84	501	281	307	55	1566	195.75 ± 162.71b	1.746	0.799
5	Madiogo pasture	02	13	84	00	263	109	416	30	917	114.63 ± 149.82b	1.374	0.689
6	Ziam 3	62	86	00	00	342	348	348	95	1281	160.13 ± 157.80b	1.581	0.769
7	Moustafari	00	124	106	77	495	176	92	400	1470	183.75 ± 171.92b	1.701	0.779
8	Patakai	144	00	22	49	117	72	73	00	477	59.63 ± 52.62b	1.655	0.791
9	Gougni	133	38	18	43	117	127	174	10	660	82.5 ± 62.16b	1.802	0.814
10	Goromo	478	02	00	00	263	260	303	19	1325	165.63 ± 184.38b	1.416	0.740
Total		1383	560	366	389	2987	2443	3096	2243	13467	1683.37 ± 1155.62	1.846	0.798
Frequencies		90	80	50	50	100	100	100	90				

Values followed by the same letters are not significantly different at the 5% level. Legend: *H*′ = Shannon index; *D*′ = Simpson index.

**Table 3:** Average seasonal density of malacofauna.

Seasons	*L. natalensis*	*B. globosus*	*B. truncatus*	*B. tropicus*	*B. pfeifferi*	*B. unicolor*	*Bithynia* sp.	*M. tuberculata*
Rainy	195 ± 75.54a	67 ± 10.39a	42.67 ± 08.14a	46 ± 05.00a	311 ± 29.46a	358 ± 48.69a	287.33 ± 63.17a	246.33 ± 45.71a
Cold dry	80.33 ± 50.36a	47.67 ± 08.62a	26.33 ± 07.64b	30.67 ± 05.68a	242.33 ± 58.53a	225.67 ± 60.18a	140.33 ± 32.25b	207.33 ± 45.39a
Hot dry	185.67 ± 24.94a	72 ± 16.70a	53 ± 08.18a	53 ± 16.52a	442.33 ± 91.13a	448.33 ± 120.34b	320 ± 75.35b	360.67 ± 96.11a

Values followed by the same letters do not show a significant difference at the 5% threshold.

**Table 4:** Physicochemical parameters of the water measured in different stations.

Parameters	Alkalinity	pH	*T* (°C)	EC (*µ*S/cm)	Depth (cm)	DO (mg/l)	DSC (ppm)
Station 1	38.88 ± 3.70	7.11 ± 0.43	27.41 ± 2.72	209.37 ± 23.3	54.78 ± 31.18	6.5 ± 0.42	180.05 ± 63.71
Station 2	36.36 ± 01.1	6.86 ± 0.23	25.74 ± 1.75	199.99 ± 26.29	33.44 ± 19.19	6.39 ± 0.44	140.23 ± 21.37
Station 3	37.20 ± 0.93	7.06 ± 0.58	26.42 ± 1.79	205.24 ± 23.69	32.28 ± 13.99	6.88 ± 0.28	151.09 ± 27.21
Station 4	31.77 ± 0.79	6.79 ± 0.31	26.65 ± 1.69	205.91 ± 21.3	48.61 ± 24.75	5.94 ± 0.19	153.32 ± 26.90
Station 5	34.09 ± 0.50	6.99 ± 0.36	26.13 ± 1.34	205.99 ± 19.42	34.78 ± 15.98	6.24 ± 0.27	151.39 ± 17.78
Station 6	36.99 ± 0.81	7.08 ± 0.29	26.97 ± 1.93	207.6 ± 21.94	27.78 ± 11.99	6.73 ± 0.28	141.54 ± 20.77
Station 7	32.77 ± 1.25	7.65 ± 0.34	26.59 ± 1.52	208.06 ± 17.51	58.52 ± 34.69	7.14 ± 0.38	160.11 ± 26.82
Station 8	29.94 ± 1.37	7.02 ± 0.32	26.69 ± 2.07	207.84 ± 31.88	36.89 ± 21.83	6.63 ± 0.22	147.13 ± 24.20
Station 9	36.67 ± 0.89	7.08 ± 0.59	26.18 ± 1.84	211.11 ± 19.09	33.33 ± 21.83	6.05 ± 0.30	159.79 ± 32.09
Station 10	34.63 ± 0.93	7.95 ± 0.59	28.14 ± 2.98	203.12 ± 28.73	71.22 ± 41.42	6.56 ± 0.20	185.20 ± 25.48
Means	34.93 ± 3.02	7.16 ± 0.53	26.69 ± 2.04	206.42 ± 22.8	43.66 ± 28.32	6.51 ± 0.46	156.98 ± 33.03

pH = hydrogen potential; *T* = temperature; EC = electric conductivity; DO = dissolve oxygen; DSC = dissolved solute content.

**Table 5:** Correlation between physicochemical parameters and mollusc populations.

Parameters	*L. natalensis*	*B. globosus*	*B. tropicus*	*B truncatus*	*B. pfeifferi*	*B. unicolor*	*Bithynia* sp.	*M. tuberculata*
Alkalinity	0.076	−0.021	0.190	0.061	0.069	**0.601**	**0.547**	**0.517**
pH	0.085	−0.141	−0.038	−0.146	**−0.208**	**−0.228**	−0.090	−0.096
Temperature	**0.439**	0.160	0.205	**0.259**	**0.388**	**0.414**	**0.265**	**0.386**
EC (*µ*S/cm)	−0.119	−0.074	−0.084	−0.077	**−0.439**	**−0.258**	−0.105	−0.171
Depth (cm)	−0.060	−0.023	−0.035	−0.004	**−0.231**	**−0.223**	−0.054	−0.077
Do (mg/l)	−0.143	−0.113	0.045	−0.138	−0.182	**−0.324**	0.015	−0.140
DSC (ppm)	−0.008	−0.078	−0.016	−0.025	**−0.248**	**−0.226**	−0.115	−0.036

Values followed by the same letters do not show a significant difference at the 5% threshold.

**Table 6:** Correlation between mollusc species and vegetation and anthropogenic activities.

Species\parameters	VC	PHH	PP	HA	Defecations
*L. natalensis*	**−0.536**	0.143	0.120	0.018	0.116
*B. globosus*	−0.161	**−0.516**	**0.408**	**0.304**	**0.406**
*B. tropicus*	−0.028	−0.195	**0.240**	0.168	**0.356**
*B. truncatus*	**−0.487**	**−0.524**	**0.590**	**0.564**	**0.478**
*B. pfeifferi*	−0.025	−0.138	**0.236**	−0.058	**0280**
*M. tuberculata*	**−0.537**	**−0.403**	**0.590**	**0.369**	0.231

Values followed by the same letters do not show a significant difference at the 5% threshold. Legend: VC = vegetation cover; PHH = proximity with human habitation; PP = proximity to pasture; HA = human activity.

**Table 7:** Larval emissions of trematodes in molluscs at the stations.

Molluscs	*L natalensis*		*B globosus*		*B tropicus*		*B truncatus*		*B. pfeifferi*		Total	
Cercariae	*F. gigantica*		*S. mansoni*		*S. mansoni*		*S. mansoni*		*S. haematobium*			
Stations	*E*(*I*)	*T*(%)	*E*(*I*)	*T*(%)	*E*(*I*)	*T*(%)	*E*(*I*)	*T*(%)	*E*(*I*)	*T*(%)	*E*(*I*)	*T*(%)
1	218 (54)	**24.77a**	101 (26)	**25.74a**	136 (19)	**13.97a**	136 (12)	**8.82a**	380 (102)	**26.84a**	971 (213)	**21.93a**
2	11 (0)	**0d**	42 (7)	**16.67a**	0	**—**	0	**—**	293 (50)	**17.06b**	346 (57)	**16.47bc**
3	151 (38)	**25.16a**	0	**—**	0	**—**	0	**—**	206 (54)	**26.21a**	357 (92)	**25.77a**
4	184 (39)	**21.19ab**	154 (42)	**27.27a**	0	**—**	84 (9)	**10.71a**	471 (76)	**16.13c**	893 (167)	**18.70b**
5	2 (0)	**0d**	13 (2)	**15.38a**	84 (11)	**13.09a**	0	**—**	263 (56)	**21.29b**	362 (69)	**19.06b**
6	62 (5)	**8.06c**	86 (23)	**26.74b**	0	**—**	0	**—**	342 (55)	**16.08c**	490 (83)	**16.94bc**
7	0	**—**	124 (40)	**32.26a**	106 (13)	**12.26a**	77 (8)	**10.39a**	467 (82)	**17.56b**	774 (143)	**18.47b**
8	144 (41)	**28.47a**	0	**—**	22 (2)	**9.1a**	49 (3)	**6.12a**	117 (21)	**17.95b**	332 (67)	**20.18ab**
9	133 (25)	**18.8b**	38 (3)	**7.89a**	18 (3)	**16.67a**	43 (1)	**2.32a**	117 (23)	**19.66b**	349 (55)	**15.76c**
10	382 (76)	**19.89ab**	2 (0)	**0a**	0	**—**	0	**—**	263 (76)	**28.9a**	647 (152)	**23.49a**
Total	1287 (278)	21.60b	560 (143)	25.53a	366 (48)	13.11c	389 (33)	8.48 d	2919 (595)	20.38b	5521 (1097)	19.87

Values followed by the same letters do not show a significant difference at the 5% threshold. Legend: *E* = examined; *I* = infected; *T* = infection rate.

**Table 8:** Seasonal variation in ringworm infestation.

Species	*L. natalensis*		*B. globosus*		*B. tropicus*		*B. truncatus*		*B. pfeifferi*		Total	
Seasons	*E*(*I*)	*T*(%)	*E*(*I*)	*T*(%)	*E*(*I*)	*T*(%)	*E*(*I*)	*T*(%)	*E*(*I*)	*T*(%)	*E*(*I*)	*T*(%)
Rainy	505 (44)	**8.71c**	201 (47)	**23.38b**	128 (15)	**11.72**	138 (15)	**10.87**	933 (156)	**16.72b**	1905 (274)	**14.38b**
Cold dry	241 (66)	**27.38b**	143 (28)	**19.58**	79 (10)	**12.66**	92 (8)	**8.69**	727 (149)	**20.49a**	1282 (259)	**20.20a**
Hot dry	541 (168)	**31.05a**	216 (68)	**31.48a**	159 (23)	**14.46**	159 (19)	**11.95**	1259 (290)	**23.03a**	2334 (564)	**24.16a**

*E* = examined; *I* = infected; *T* = infection rate. Values followed by the same letters are not significantly different at the 5% level.

**Table 9:** Relationships between cercarial emissions and physicochemical parameters of water.

Species	Cercariae	Alkalinity	PH	Temperature (°C)	EC (*µ*S/cm)	Depth (cm)	DO (mg/l)	DSC (ppm)
*L. natalensis*	*F. gigantica*	0.018	0.026	**0.261**	**0.240**	0.016	0.027	0.002
*B. globosus*	*S. haematobium*	0.000	0.015	**0.055**	0.022	0.009	0.006	0.029
*B. tropicus*	*S. haematobium*	0.035	0.004	0.034	0.020	0.006	0.005	0.000
*B. truncates*	*S. haematobium*	0.000	0.016	0.041	0.012	0.001	0.006	0.001
*B. pfeifferi*	*S. mansoni*	**0.069**	0.027	**0.228**	**0.237**	0.030	0.023	0.013

Values in bold are significantly different from 0 at the alpha = 0.05 significance level.

**Table 10:** Correlations between cercarial emissions and vegetation and anthropogenic activities.

Molluscs	Cercariae	VC	PHH	PP	HA	Defecations
*L. natalensis*	*F. gigantica*	**−0.573**	0.075	**0.226**	0.126	0.128
*B. globosus*	*S. haematobium*	−0.136	**−0.391**	**0.281**	**0.212**	**0.404**
*B. tropicus*	*S. haematobium*	−0.055	−0.190	**0.270**	0.167	**0.297**
*B. truncates*	*S. haematobium*	**−0.304**	**−0.300**	**0.401**	**0.298**	**0.337**
*B. pfeifferi*	*S. mansoni*	−0.152	0.008	**0.257**	−0.092	0.182

Values in bold are significantly different from 0 at the alpha = 0.05 significance level.

## Data Availability

The data of the study are available from the corresponding author upon request.
